# Dietary fatty acid intake affects the risk of developing bone marrow lesions in healthy middle-aged adults without clinical knee osteoarthritis: a prospective cohort study

**DOI:** 10.1186/ar2688

**Published:** 2009-05-08

**Authors:** Yuanyuan Wang, Miranda L Davies-Tuck, Anita E Wluka, Andrew Forbes, Dallas R English, Graham G Giles, Richard O'Sullivan, Flavia M Cicuttini

**Affiliations:** 1 Department of Epidemiology and Preventive Medicine, School of Public Health and Preventive Medicine, Monash University, Alfred Hospital, Commercial Road, Melbourne, VIC 3004, Australia; 2 Baker Heart and Diabetes Research Institute, Commercial Road, Melbourne, VIC 3004, Australia; 3 Centre for Molecular, Environmental, Genetic and Analytic Epidemiology, School of Population Health, University of Melbourne, Swanston Street, Carlton, VIC 3053, Australia; 4 Cancer Epidemiology Centre, The Cancer Council Victoria, Rathdowne Street, Carlton, VIC 3053, Australia; 5 MRI Unit, Symbion Imaging, Epworth Hospital, Bridge Road, Richmond, VIC 3121, Australia

## Abstract

**Introduction:**

Fatty acids have been implicated in osteoarthritis (OA), yet the mechanism by which fatty acids affect knee structure and consequently the risk of knee OA has not been fully elucidated. Higher intakes of fatty acids have been shown to be associated with the risk of bone marrow lesions (BMLs) in a healthy population. The aim of this study was to examine the association between fatty acid consumption and the incidence of BMLs in healthy middle-aged adults without clinical knee OA.

**Methods:**

Two hundred ninety-seven middle-aged adults without clinical knee OA underwent magnetic resonance imaging (MRI) of their dominant knee at baseline. BMLs were assessed. Of the 251 participants with no BMLs in their knee at baseline, 230 underwent MRI of the same knee approximately 2 years later. Intakes of fatty acids were estimated from a food frequency questionnaire.

**Results:**

Increased consumption of saturated fatty acids was associated with an increased incidence of BMLs over 2 years after adjusting for energy intake, age, gender, and body mass index (odds ratio of 2.56 for each standard deviation increase in dietary intake, 95% confidence interval 1.03 to 6.37, *P *= 0.04). Intake of monounsaturated or polyunsaturated fatty acids was not significantly associated with the incidence of BMLs.

**Conclusions:**

Increased fatty acid consumption may increase the risk of developing BMLs. As subchondral bone is important in maintaining joint integrity and the development of OA, this study suggests that dietary modification of fatty acid intake may be one strategy in the prevention of knee OA which warrants further investigation.

## Introduction

Nutritional factors have been shown to be important in the maintenance of bone and joint health [1]. In particular, fatty acids have been implicated in osteoarthritis (OA) [2,3]. Elevated levels of fat and n-6 polyunsaturated fatty acids have been found in OA bone [2], whereas n-3 polyunsaturated fatty acids have been shown to alleviate progression of OA through an effect on the metabolism of articular cartilage [3]. Although dietary supplementation with polyunsaturated fatty acids has been shown to decrease bone turnover and increase bone mineral density [4], the finding that a higher ratio of n-6 to n-3 polyunsaturated fatty acids is associated with lower bone mineral density at the hip [5] suggests the important role of relative amounts of these polyunsaturated fatty acids in preserving skeletal integrity in older age.

However, the mechanism by which polyunsaturated fatty acids affect the knee structure and consequently the risk of knee OA has not been fully elucidated. We have recently shown that higher intakes of monounsaturated, total, and n-6 polyunsaturated fatty acids were associated with an increased prevalence of bone marrow lesions (BMLs) in a healthy population without clinical knee OA [6]. BMLs have been associated with structural changes of disease severity, including increased cartilage defects, tibial plateau area, loss of cartilage, and joint space narrowing, suggesting that they play a role in the pathogenesis of OA [7-9]. However, there are no longitudinal studies examining the role of fatty acids on incident BMLs in either healthy or OA populations. Therefore, the aim of this study was to examine the association between intakes of different types of fatty acids and the incidence of BMLs in healthy, community-based, middle-aged men and women with no clinical knee OA.

## Materials and methods

### Subjects

This study was conducted within the Melbourne Collaborative Cohort Study (MCCS), a prospective cohort study of 41,528 Melbourne, Australia residents who were 40 to 69 years old at recruitment (1990 to 1994) [10]. Participants for the current study were recruited from within the MCCS between 2003 and 2004 as previously described [6]. Briefly, participants were eligible if they were between 50 and 79 years old without any of the following exclusion criteria: a clinical diagnosis of knee OA as defined by American College of Rheumatology criteria [11], knee pain lasting for more than 24 hours in the last 5 years, a previous knee injury requiring non-weight-bearing treatment for more than 24 hours or surgery (including arthroscopy), or a history of any form of arthritis diagnosed by a medical practitioner. A further exclusion criterion was a contraindication to magnetic resonance imaging (MRI), including pacemaker, metal sutures, presence of shrapnel or iron filings in the eye, or claustrophobia. The study was approved by The Cancer Council Victoria's Human Research Ethics Committee and the Standing Committee on Ethics in Research Involving Humans of Monash University. All participants gave written informed consent.

### Anthropometric and dietary data

Height was measured using a stadiometer with shoes removed. Weight was measured using electronic scales with bulky clothing removed. Body mass index (BMI) (weight/height^2^, kg/m^2^) was calculated. At MCCS baseline, questionnaires covered demographic data and diet (via a 121-item food frequency questionnaire developed from a study of weighed food records [12]). Fatty acid intakes were calculated from the food frequency questionnaire using Australian food composition data and were adjusted for energy intake [13].

### Magnetic resonance imaging and the measurement of bone marrow lesions

Each subject had an MRI performed on the dominant knee, determined from kicking preference [14], at baseline and approximately 2 years later. Knees were imaged on a 1.5-T whole-body magnetic resonance unit (Philips Medical Systems, Eindhoven, The Netherlands) using a commercial transmit-receive extremity coil, with coronal T_2_-weighted fat-saturated acquisition as previously described [9]. BMLs were defined as areas of increased signal intensity adjacent to subcortical bone present in either the medial or lateral, distal femur or proximal tibia [9]. Two trained observers, blinded to patient characteristics and sequence of images, together assessed the presence of lesions for each subject. The baseline and follow-up images were assessed unpaired. A lesion was defined as present if it appeared on two or more adjacent slices and encompassed at least one quarter of the width of the tibial or femoral cartilage being examined from coronal images, equivalent to a 'large BML' as described by Felson and colleagues [9]. The reproducibility for determination of BMLs was assessed using 60 randomly selected knee MRIs (κ value 0.88, *P *< 0.001).

### Statistical analyses

The descriptive statistics of the characteristics of study participants were tabulated. Participants with self-reported total energy intakes in the top or bottom 1% of the gender-specific distributions were excluded. A BML was defined as incident if it was present at follow-up in the knees without BMLs at baseline. Logistic regression models were constructed to explore the relationship between fatty acid intakes and incident BMLs after adjusting for potential confounders of age, gender, BMI, and energy intake. Intake of fatty acids was standardised so that the coefficients represent the effect of an increment of one standard deviation (SD) in intake. *P *values of less than 0.05 were considered to be statistically significant. All analyses were performed using the SPSS statistical package (standard version 15.0.0; SPSS Inc., Cary, NC, USA).

## Results

Two hundred ninety-seven subjects entered the study, and four subjects were excluded due to having energy intakes in the top or bottom 1% of the gender-specific distributions. Of the 251 participants who did not have a BML at baseline, 230 (92%) completed the 2-year follow-up. Participants lost to follow-up had a higher BMI (*P *= 0.04) compared with those who completed follow-up. There were no significant differences in consumption of saturated (*P *= 0.56), monounsaturated (*P *= 0.59), or polyunsaturated (*P *= 0.75) fatty acids between the two groups. Thirty-two subjects developed BMLs at follow-up. Participants who developed BMLs had a higher BMI (mean [SD] 27.9 [5.3] versus 25.4 [3.8] kg/m^2^, *P *= 0.02) and higher energy intake-adjusted saturated fatty acid consumption (mean [standard error] 35.7 [1.2] versus 33.0 [0.5] g/day, *P *= 0.03) when compared with those who did not. There was no significant difference in terms of the energy intake-adjusted consumption of monounsaturated and polyunsaturated fatty acids (Table 1).

**Table 1:** Characteristics of study participants with no bone marrow lesions at baseline

	Incident BMLs (n = 32)	Without incident BMLs (n = 198)	*P *value^a^
Age, years	57.6 (5.8)	57.7 (5.0)	0.91
Number of females (percentage of females)	23 (72%)	120 (61%)	0.22^b^
Body mass index, kg/m^2^	27.9 (5.3)	25.4 (3.8)	0.02
Energy intake, kJ/d	8,822 (3,019)	9,293 (3,063)	0.42
Saturated fatty acid, g/day	35.7 (1.2)	33.0 (0.5)	0.03^c^
Monounsaturated fatty acids, g/day	29.3 (0.9)	27.9 (0.4)	0.14^c^
Polyunsaturated fatty acids, g/day	12.7 (0.7)	12.5 (0.3)	0.76^c^
n-3 polyunsaturated fatty acids, g/day	1.2 (0.05)	1.2 (0.02)	0.60^c^
n-6 polyunsaturated fatty acids, g/day	11.3 (0.6)	11.4 (0.3)	0.92^c^
n-6/n-3 ratio	9.6 (0.5)	9.7 (0.2)	0.82^c^

Data are presented as mean (standard deviation) unless otherwise stated. ^a^*P *value for comparisons between two groups using independent samples *t *test, ^b^chi-square test, or ^c^one-way analysis of covariance after adjusting for energy intake. BMLs, bone marrow lesions.

Although there was no significant association between fatty acid consumption and the incidence of BMLs over 2 years in univariate analysis, higher consumption of saturated fatty acids was significantly associated with an increased risk of developing BMLs after adjusting for energy intake (Table 2, model 1). For each SD increase in dietary intake of saturated fatty acids, the risk of developing BMLs over 2 years increased 2.62-fold (95% confidence interval [CI] 1.11 to 6.17). This relationship persisted after further adjusting for age, gender, and BMI (odds ratio 2.56, 95% CI 1.03 to 6.37) (Table 2, model 2). No significant association between consumption of monounsaturated or polyunsaturated fatty acids or n-6/n-3 ratio and incident BMLs was found in multivariate analyses (Table 2).

**Table 2:** Relationship between fatty acid intake and incidence of bone marrow lesions

	Univariate analysis, OR (95% CI)	*P *value	Model 1 Multivariate analysis, OR (95% CI)^a^	*P *value	Model 2 Multivariate analysis, OR (95% CI)^b^	*P *value
Saturated fatty acids	1.08 (0.72–1.60)	0.73	2.62 (1.11–6.17)	0.03	2.56 (1.03–6.37)	0.04
Monounsaturated fatty acids	1.01 (0.66–1.52)	0.98	2.10 (0.81–5.47)	0.13	1.99 (0.75–5.31)	0.17
Polyunsaturated fatty acids	0.94 (0.62–1.42)	0.77	1.10 (0.64–1.90)	0.74	1.10 (0.62–1.96)	0.74
n-6 polyunsaturated fatty acids	0.88 (0.57–1.35)	0.55	0.98 (0.56–1.70)	0.93	0.98 (0.55–1.76)	0.96
n-3 polyunsaturated fatty acids	0.81 (0.53–1.25)	0.34	0.85 (0.46–1.56)	0.60	0.85 (0.45–1.61)	0.62
n-6/n-3 ratio	0.94 (0.63–1.38)	0.74	0.96 (0.65–1.41)	0.82	0.93 (0.61–1.42)	0.74

^a^Model 1: odds ratio for development of tibiofemoral bone marrow lesions for each increase of 1 standard deviation in the respective fatty acid intake after adjusting for energy intake. ^b^Model 2: odds ratio for development of tibiofemoral bone marrow lesions for each increase of 1 standard deviation in the respective fatty acid intake after adjusting for energy intake, age, gender, and body mass index. CI, confidence interval; OR, odds ratio.

From MCCS baseline when dietary fatty acid intake data were collected during 1990 to 1994 to the inception of current study when baseline MRI was performed in 2003 to 2004, the weight of participants increased by a mean of 2.1 kg (SD 5.2 kg). After adding weight gain to model 2, consumption of saturated fatty acids persisted to be positively associated with incident BMLs (odds ratio 2.54, 95% CI 1.01 to 6.39).

There was no evidence that BMI modified the association between energy intake-adjusted dietary saturated fatty acid consumption and incident BMLs when an interaction term for BMI category × saturated fatty acid intake was included in the logistic model with adjustment for energy intake. The *P *value was 0.64 when BMI was categorised as less than 25 kg/m^2^, 25 to 30 kg/m^2^, and greater than or equal to 30 kg/m^2^.

## Discussion

In a population of healthy middle-aged adults with no clinical knee OA, we found that higher intake of saturated, but not monounsaturated or polyunsaturated, fatty acids or that the n-6/n-3 ratio was associated with an increased likelihood of developing BMLs over 2 years. This is the first longitudinal study presenting a relationship between dietary fatty acid intake and the incidence of BMLs. We have previously shown in a cross-sectional study that increased dietary intake of monounsaturated and n-6, but not n-3, polyunsaturated fatty acids were associated with an increased risk of having BMLs in a healthy population without clinical knee OA [6]. When this population was followed up for 2 years, we found an association between higher saturated fatty acid intake and increased likelihood of developing BMLs over 2 years. Although the mechanism for the discrepancy in terms of the type of fatty acid consumption observed between the previous cross-sectional study and the current prospective cohort study is unclear, the adverse effect of saturated fatty acids on the incidence of BMLs may be attributed to a vascular effect. Saturated fatty acid intake has been associated with atherosclerosis and cardiovascular disease [15]. There are no previous studies identifying a relationship between saturated fatty acid intake and the risk of OA. Recently, it has been suggested that atheromatous vascular disease may be important in the progression of OA [16] and that subchondral ischaemia may be a mechanism by which vascular pathology plays a role in the initiation and/or progression of OA [17]. The findings of this study therefore suggest that vascular disease in subchondral bone may play a role in the pathogenesis of OA via BMLs.

There is mounting evidence that BMLs play a role in the pathogenesis of OA [7-9]. It has been demonstrated that BMLs are associated with the presence of cartilage defects in healthy asymptomatic populations with no history of significant knee pain or injury and that risk factors for OA such as age, height, and BMI also affect the prevalence of BMLs [18,19]. Moreover, the presence of BMLs predicts the progression of cartilage defects and loss of cartilage volume over 2 years in longitudinal studies [20,21]. These findings suggest that BMLs may be associated with an increased risk of knee OA. This study demonstrates an increased incidence of BMLs associated with increased saturated fatty acid intake in a healthy population and suggests that modifying diet may be one such way to reduce the development and subsequent burden of OA.

This study has a number of potential limitations. First, this study examined a healthy, community-based population selected on the criterion of having no knee pain or injury and therefore the results may not be generalisable to symptomatic populations or people who have injured their knees. However, the findings of our study can be generalised to populations that would be targeted by primary prevention strategies. Second, whilst the dietary intake of fatty acids was measured in a valid fashion [22], this was based on a single measure of nutrient intakes 10 years earlier. Although significant underreporting of fat intake is likely [23], absolute intake of dietary fat tends to remain stable [24,25]. While nutritional data collected 10 years earlier may have resulted in some misclassification of exposure, such misclassification is likely to have been non-differential in relation to knee structure since only subjects with no history of knee symptoms or injury were included, thereby tending to underestimate the strength of any observed associations. In the current study, we did not measure knee alignment, which has been shown to be associated with BMLs [9].

## Conclusions

The findings of this study suggest that increased fatty acid consumption may increase the risk of developing BMLs in a healthy population without clinical knee OA. As subchondral bone is important in maintaining joint integrity and the development of OA, this study suggests that dietary modification of fatty acid intake may be one strategy in the prevention of knee OA which warrants further investigation.

## Abbreviations

BMI: body mass index; BML: bone marrow lesion; CI: confidence interval; MCCS: Melbourne Collaborative Cohort Study; MRI: magnetic resonance imaging; OA: osteoarthritis; SD: standard deviation.

## Competing interests

The authors declare that they have no competing interests.

## Authors' contributions

YW participated in the design of the study, performed the statistical analysis and the interpretation of data, and drafted the manuscript. MLD-T performed the measurement of bone marrow lesions, participated in the statistical analysis and the interpretation of data, and drafted the manuscript. AEW participated in the interpretation of data and reviewed the manuscript. AF helped in the statistical analysis and reviewed the manuscript. DRE and GGG participated in the design of the study and the acquisition of data and reviewed the manuscript. RO provided technical support and reviewed the manuscript. FMC participated in the design of the study, helped in the interpretation of data, and reviewed the manuscript. All authors read and approved the final manuscript.
